# DetectaWeb-Distress Scale: A Global and Multidimensional Web-Based Screener for Emotional Disorder Symptoms in Children and Adolescents

**DOI:** 10.3389/fpsyg.2021.627604

**Published:** 2021-02-15

**Authors:** Jose A. Piqueras, Mariola Garcia-Olcina, Maria Rivera-Riquelme, Agustin E. Martinez-Gonzalez, Pim Cuijpers

**Affiliations:** ^1^Department of Health Psychology, Faculty of Social and Health Sciences, Center for Applied Psychology, Miguel Hernandez University, Elche, Spain; ^2^Department of Developmental Psychology and Didactics, Faculty of Education, University of Alicante, San Vicente del Raspeig, Spain; ^3^Department of Clinical Psychology, Vrije Universiteit Amsterdam, Amsterdam, Netherlands; ^4^The Netherlands & EMGO Institute for Health and Care Research, Amsterdam, Netherlands

**Keywords:** emotional, distress, anxiety, depression, screener, children and adolescents, web-based, detectaweb-distress

## Abstract

Emotional disorder symptoms are highly prevalent and a common cause of disability among children and adolescents. Screening and early detection are needed to identify those who need help and to improve treatment outcomes. Nowadays, especially with the arrival of the COVID-19 outbreak, assessment is increasingly conducted online, resulting in the need for brief online screening measures. The aim of the current study was to examine the reliability and different sources of validity evidence of a new web-based screening questionnaire for emotional disorder symptoms, the DetectaWeb-Distress Scale, which assesses mood (major depression and dysthymic disorder), anxiety (separation anxiety, generalized anxiety, social phobia, panic disorder/agoraphobia, and specific phobia), obsessive–compulsive disorder, post-traumatic stress disorder, suicidality (suicidal ideation, plans, and attempts), and global distress. A total of 1,499 participants (aged 8–18) completed the DetectaWeb-Distress Scale and specific questionnaires for emotional disorder symptoms, suicidal behaviors, and well-being through a web-based survey. Results indicated that a structural model of 10 correlated factors fits reasonably better in comparison to the remaining models; measurement invariance for age and gender; good internal consistency (McDonald's ω ranging from 0.65 to 0.94); and significant positive correlation with other measures of anxiety, depression, PTSD, or distress, and negative correlation with well-being measures, displaying support for convergent-discriminant validity. We also found that girls scored higher than boys on most of the subscales, and children had higher scores for social anxiety, specific phobia, panic disorder, and obsessive–compulsive symptoms, whereas adolescents scored higher on depressive symptoms, suicidality, and generalized anxiety, but the effect sizes were small to medium for all comparisons. The DetectaWeb-Distress Scale is a valid, innovative, and useful online tool for the screening and evaluation of preventive programs for mental health in children and adolescents.

## Introduction

The last years of childhood and adolescence are key stages from the human developmental point of view, in which many physical, cognitive, and psychosocial changes take place, overlapping with the acquisition of new roles and responsibilities (Susman and Dorn, 2009; Steinberg, 2017). Therefore, an important development and maturation of the individual takes place, acquiring a wide and significant repertoire of personal skills that will be key to personal success later in adult life. All of these changes and demands may also be stressful, and individuals can feel emotionally overwhelmed, making them at risk of reduced mental health. Consequently, this stage is an extremely sensitive period for the development of mental health problems.

The World Health Organization report titled “Health for the World's Adolescents: A Second Chance in the Second Decade” suggests that anxiety and related disorders such as other emotional disorders (i.e., depression) are some of the most common mental disorders and most frequent causes of disease and disability in children and adolescents (World Health Organization, 2014a). Furthermore, suicide is the second leading cause of death in adolescents (World Health Organization, 2014b). More specifically, according to a recent meta-analytic review, global prevalence rates of these disorders in youth are 6.5% for anxiety and 2.6% for depressive disorders (Polanczyk et al., 2015), with a marked comorbidity between the two disorders (Cummings et al., 2014; Al-Asadi et al., 2015). Additionally, several studies among the general population of young people show that common mental disorders are one of the main risk factors for suicidal ideation and behavior (OR = 2.07–10.06) (Gili et al., 2019).

Concerning Spain, in example, Ezpeleta et al. (2007) found that between 30 and 60% of preadolescents and between 30 and 50% of adolescents presented some mental disorder, with anxiety and depression disorders as some of the most frequent disorders. Later, in 2019, Canals et al. found a prevalence of any anxiety disorder of 11.8%, with high rates of comorbidity with depression and other anxiety disorders, low use of professional support (33.3%), and high persistence of diagnosis in a 2-year follow-up (52.9%). More recently, the increase of psychological and behavioral changes, especially emotional symptoms, in Spanish children and adolescents during the early phase of COVID-19 quarantine has been reported (Francisco et al., 2020). Also, this presence of emotional symptoms, among others, at subclinical level raise the risk of subsequent development of mental disorder (Fonseca-Pedrero et al., 2020). However, the approach to adolescent mental health must contemplate not only a psychopathological view, but both the presence of difficulties and strengths (i.e., Piqueras et al., 2019; Rivera-Riquelme et al., 2019; Fonseca-Pedrero et al., 2020).

There are at least four reasons for the need for valid and reliable screening instruments for children and adolescent mental health problems (Stiffler and Dever, 2015; Tran et al., 2019).

First, it is necessary and beneficial for clinical practice to evaluate both emotional disorders and symptoms and related conditions in children and adolescents, when a first diagnostic approach is made and also to detect these symptoms in the general population (Ebesutani et al., 2012). Second, any approach to the evaluation of these disorders entails the problem of the mental health professional's lack of time to perform a diagnostic assessment, with the most common practice being the use of self-report tests to screen for these symptoms (Ebesutani et al., 2012). Third, self-report instruments have proven to be the first choice for the screening and detection of anxiety and depression with undoubted advantages over other techniques such as clinical interviews or observation techniques (Southam-Gerow and Chorpita, 2007).

Furthermore, assessment of emotional disorders is conducted increasingly online, mainly also employing self-report questionnaires (Kendrick and Pilling, 2012). Online assessment offers other advantages for participants and researchers, due to the fact that it reduces the load and allows for greater disclosure (Mogle, 2015). Recently, van Ballegooijen et al. (2016) summarized the psychometric properties of diverse online instruments evaluating anxiety and depression disorders. According to this review, there are online instruments for depression, anxiety, OCD, or PTSD for adolescents with good psychometric properties, reporting Cronbach's α ranging from 0.73 to 0.93 and evidence of convergent and criterion validity (Cuijpers et al., 2008; Keeley and Storch, 2009; Zlomke, 2009; Haavet et al., 2011). However, the review did not include any studies with children, and those found with adolescent samples were scarce. In recent times, especially with the arrival of the COVID-19 outbreak, different studies have noted the need to use online validated multi-informant and multi-problem approaches during and after home confinement (Espada et al., 2020).

Concerning the existing instruments, we shall attempt a brief description of the available tools for children's mental health professionals. According to Stiffler and Dever (2015), there are a considerable number of them, ranging from broadband multidimensional measures, through specific screenings for single vs. for multiple disorders, to those including only one indicator of overall distress.

The international community has several broad-spectrum screening measures that try to evaluate both negative and positive aspects of functioning, including an overall score of emotional problems [i.e., the Behavior Assessment System for Children and Adolescents (BASC; Reynolds and Kamphaus, 2004); the Achenbach System of Empirically Based Assessment (ASEBA; Achenbach and Rescorla, 2001, 2007); the Y-PSC-17 (Jellineck et al., 1988); the Strengths and Difficulties Questionnaire (SDQ; Goodman, 1997); or the Child and Adolescent Assessment System (SENA; Fernández-Pinto et al., 2015a,b)]. However, none of these broad-spectrum measures specifically assesses each type of anxiety, depression, and related disorder symptoms, such as PTSD, OCD, or suicidality.

Beyond these multi-component and broad-spectrum tools, there are also specific measures for only anxiety or depressive disorder symptoms. Therefore, different measures—such as the Multidimensional Anxiety Scale for Children (MASC; March et al., 1997), Children's Spence Anxiety Scale (SCAS; Spence, 1997), the Screen for Child Anxiety Related Emotional Disorders-Revised (SCARED-R; Muris et al., 1998), or the most recently published Youth Anxiety Measurement for the *Diagnostic and Statistical Manual of Mental Disorders, fifth edition* (DSM-5) (YAM-5, Muris et al., 2017), among others—provide valuable information not only about anxiety levels in general but also about the type of anxiety symptoms experienced by children and adolescents. Thus, these assessment tools allow examining the different types of anxiety disorders and potential comorbidity between them (Spence, 2018).

As regards depression self-reports for children and adolescents, a systematic review and meta-analysis of reliability, validity, and diagnostic utility (Stockings et al., 2015) showed that commonly used depression symptom rating scales, such as the Children's Depression Inventory (CDI, Kovacs, 1981), the Beck Depression Inventory (BDI, Beck et al., 1961), the Center for Epidemiologic Studies-Depression Scale (CES-D; Radloff, 1977), and the Reynolds Adolescent Depression Scale (RADS; Reynolds, 1986), are reliable measures of depressive symptoms among children and adolescents. However, they only provide overall scores of depression or components of depressive symptoms.

Beyond these specific tools, there are also comprehensive tests for the assessment of both anxiety and depression, such as the Revised Childhood Anxiety and Depression Scale (RCADS; Chorpita et al., 2000) and the short version RCADS-30 (Sandin et al., 2010). A recent meta-analysis indicates that the internal consistency of the different versions of the RCADS are equally high and equivalent to each other (Piqueras et al., 2017b). However, none of these versions allow one to assess some anxiety disorders, such as specific phobia, other related emotional disorders, such as dysthymia or PTSD, or suicidality.

Finally, different authors have developed measures to uniquely apprehend overall psychological distress, such as the well-known Kessler Psychological Distress Scale (Kessler et al., 2002) or the Social Emotional Distress Scale (SEDS; Dowdy et al., 2018), among others. According to Kessler et al. (2002), dimensional measures of global psychological distress have emerged, which are important to distinguish community cases based on severity rather than purely on diagnosis. From this wider framework in the assessment of mental disorders, some authors consider that subjective distress is determined largely by the presence of emotional or internalizing symptoms: anxiety, depression (Mewton et al., 2016; Dowdy et al., 2018), and suicidality (van Ballegooijen et al., 2016). Accordingly, internalizing disorders can be differentiated in two sets: distress or misery disorders such as generalized anxiety disorder (GAD), post-traumatic stress disorder (PTSD), major depression (MD), and dysthymic disorder (DD); and fear/anxiety disorders (such as panic and phobias) (Krueger, 1999; Watson, 2005; Clark and Watson, 2006). In fact, since this framework, anxiety, anxiety-related disorders such as OCD or PTSD, and depressive disorders can be collapsed into an overcharging class of “internalizing” or emotional disorders. From a broader point of view, according to relevant authors in this field (Krueger, 1999; Kessler et al., 2002; Watson, 2005; Clark and Watson, 2006), psychological distress can be conceptualized as the presence of symptoms of some of the emotional disorders without differentiating them. In fact, some authors have used the expression “depression-anxiety disorders spectrum,” “emotional disorders spectrum,” or “emotional disorders continuum” that would include different nosological entities such as misery and fear disorders, and highlighting that all these anxiety and depression-related disorders would share an internalizing factor (i.e., Gorman, 1996; Watson, 2005). In fact, a large consortium of researchers has more recently proposed the Hierarchical Taxonomy of Psychopathology (HiTOP) as an alternative to traditional categorical classifications (Kotov et al., 2017). This theoretical system has been articulated to address the limitations currently plaguing psychiatry, such as the DSM-5. Therefore, this model is a dimensional alternative to traditional nosologies for mental disorders. Their basic characteristics are (1) to consider mental health as a spectrum, that is, as a continuum between psychopathology and normality, or in other words, to consider psychopathology not as an entity in itself, but as a spectrum where different problems may share similar characteristics, and (2) to simplify the diagnostic classification, since there is a great comorbidity or overlap between disorders and this hierarchical proposal solves these difficulties.

Therefore, considering the above, our team created a new measure, the DetectaWeb-Distress Scale, the first web-based screening questionnaire for the assessment of some of the most common mental disorders among children and adolescents, such as specific subtypes of anxiety, and some of the more common anxiety-related emotional disorders such as depression, OCD, PSTD, suicidality, and overall psychological distress (Garcia-Olcina et al., 2014, 2017; Piqueras et al., 2017a, 2020). The main reason for the development of this new instrument was the need for a new specific screening instrument to detect the main emotional disorders, which should be brief, as adolescents undergo screening more easily if it is short, fast, and easy to read, according to Cuijpers et al. (2009). Furthermore, this new questionnaire is a step forward in terms of the assessment of some of the main emotional disorders through the Internet, having a potential usefulness in different fields such as child and adolescent psychopathology and clinical psychology through new technologies (i.e., for epidemiological and screening studies, diagnosis and treatment, treatment evaluation research, etc.).

The aim of the present study was the evaluation of the psychometric properties of the DetectaWeb-Distress Scale. We expected the following findings concerning different sources of validity evidence: (a) a factor structure of 10 factors to be evaluated; (b) measurement equivalence for gender and age; (c) gender and age differences in scores, such that girls and adolescents would score higher than boys and children in the total and partial scores; (d) good internal consistency reliability for the total scale and subscales; and (e) adequate validity in terms of positive and significant correlations between the total score and the subscale scores and with other patient-reported outcome measure for internalizing problems, and negative and significant correlations with different constructs.

## Materials and Methods

### Participants

In this study, we used a convenience sampling method. We selected eight centers of primary and secondary education through a random cluster sampling of the main counties (north, central, and south) of the province of Alicante (Valencian Community, Spain). In order to ensure that all geographic areas of the province were represented, one public and one semi-private school per county were randomly selected.

The initial sample consisted of 1,523 children and adolescents, of whom 24 were eliminated because either they did not attend school the day the survey was applied (*n* = 13) or they were over 18 years old (*n* = 11). The general inclusion criteria were the following: (a) ages 8 to 18 years and (b) being enrolled from 3rd grade of elementary education to 2nd grade of higher education. Exclusion criteria were as follows: (a) insufficient knowledge of Spanish language; (b) parents or guardians did not provide informed consent to the study, or children older than 12 did not give informed consent (compulsory according to Spanish law); and (c) students who did not attend class the day of the assessment [for further details concerning the sampling procedure, sample features, inclusion/exclusion criteria, and recruitment plan to assure sample representativeness, please see Piqueras, Garcia-Olcina et al. (2017)].

The final sample consisted of 1,499 children and adolescents (754 males) between 8 and 18 years old (*M* = 12.70, SD = 2.78). Most of the sample was born in Spain (93.6%). The Family Affluence Scale (Currie et al., 1997) indicated that 14.3% had a low socioeconomic status (SES), 44.1% had an intermediate SES, and 41.6% had a high SES. The distribution of children and adolescents by age and gender is presented in Table 1.

**Table 1:** Number and percentages of children and adolescents by age and gender.

		**Age**											
		**8** ***n* (%)**	**9** ***n* (%)**	**10** ***n* (%)**	**11** ***n* (%)**	**12** ***n* (%)**	**13** ***n* (%)**	**14** ***n* (%)**	**15** ***n* (%)**	**16** ***n* (%)**	**17** ***n* (%)**	**18 *n* (%)**	**Total**
Gender	Female	58 (7.80)	68 (9.10)	83 (11.10)	74 (9.90)	57 (7.70)	82 (11.00)	99 (13.30)	70 (9.40)	83 (11.10)	55 (7.40)	16 (2.10)	745
	Male	52 (6.90)	70 (9.30)	67 (8.90)	73 (9.70)	87 (11.50)	96 (12.70)	86 (11.40)	83 (11.0)0	73 (9.70)	50 (6.60)	17 (2.30)	754
	Total	110 (7.30)	138 (9.20)	150 (10.00)	147 (9.80)	144 (9.60)	178 (11.90)	185 (12.30)	153 (10.20)	156 (10.40)	105 (7.00)	33 (2.20)	1499

Regardless of using convenience sampling, the adoption of the random cluster sampling method ensured the heterogeneity and representativeness of the sample. Thus, chi-square tests indicated that there was no interdependence between gender and age (χ^2^ = 12.29, *p* = 0.26), between gender and nationality (χ^2^ = 7.25, *p* = 0.29), or between gender and SES (χ^2^ = 0.70, *p* = 0.70).

### Instruments

#### Sociodemographic Factors

*The Family Affluence Scale* (*FAS;* Currie et al., 1997) was used to measure SES. Scores from zero to seven represent categories of low (0–3), intermediate (4–5), or high (6–7) family wealth. The FAS has shown good criterion and construct validity in previous studies with adolescents (Boyce et al., 2006).

#### Internalizing Disorder Symptoms

The DetectaWeb-Distress Scale (Garcia-Olcina et al., 2014) is a web-based screening questionnaire created by our team. It consists of 30 items (3 items per subscale) that assess anxiety disorders, such as separation anxiety disorder (SAD), generalized anxiety disorder (GAD), specific phobia (SP), panic disorder/agoraphobia (Pd/Ag), and social phobia (SoPh); some of the main anxiety-related disorders, such as post-traumatic stress disorder (PTSD) and obsessive–compulsive disorder (OCD); mood disorders, such as major depression (MD) and dysthymic disorder (DD); suicidality (S—suicidal ideation, plans, and attempts); and a total score indicating global distress or emotional symptomatology. It is rated on a Likert-type response format (0–3). A pilot study with adolescents between 14 and 18 years old provided initial support for the reliability and validity to assess anxiety, depression, and suicidal thoughts and behaviors (Garcia-Olcina et al., 2014). The measure had good internal consistency for the Global Distress Scale (α = 0.87) and adequate correlations with related measures of anxiety and depression (RCADS: *r* = 0.40–87). Additionally, another study showed that, preliminarily, this measure is a reliable, valid, and useful tool to assess emotional disorders in a clinical sample (Garcia-Olcina et al., 2017), and a recent published work found that the DetectaWeb-Distress Scale is a useful measure from a diagnostic point of view, since it discriminates between people with anxiety, depression, and suicide disorders and those who do not suffer from them, presenting ROC values around 0.80 and good sensitivity and specificity for detecting the main emotional disorders (Piqueras et al., 2020). Specifically, sensitivity and specificity values of DetectaWeb-Distress total score for anxiety, depression, emotional (any anxiety or depression), and internalizing (any of them, including anxiety, depression, OCD, or PTSD) diagnosed disorders were 0.75/0.76, 0.81/0.72, 0.73/0.77, and 0.73/0.78, respectively. Additionally, a score of 25 (range = 0–90) for the total score was found to be the recommended cutoff score for a positive diagnosis. Concerning the specific subscales, the sensitivity and specificity estimates were as follows: 0.86/0.68 (SAD), 62/0.77 (GAD), 0.83/0.84 (SP), 75/0.63 (Pd/Ag), 0.62/0.82 (SoPh), 1.00/0.81 (OCD), 0.67/0.89 (PTSD), 0.75/0.96 (MD), 0.64/0.84 (DD), and 0.50/0.99 (S). The recommended cutoff scores for a positive diagnosis were respectively 4, 6, 4, 2, 5, 3, 4, 6, 4, and 4 (range = 0–9).

The *Revised Child Anxiety and Depression Scale, 30-item version RCADS-30* (Sandin et al., 2010) is a reduced version of the RCADS (Chorpita et al., 2000; Sandin et al., 2009). It comprises 30 items and six subscales for evaluating symptoms of the following disorders: Pd, SoPh, SAD, GAD, OCD, and MD. Response options range from 0 (*never*) to 3 (*always*). The scale showed excellent psychometric properties in international studies and with Spanish samples (Piqueras et al., 2017b). In this sample, the McDonald's ω were as follows: Pd (0.78), SoPh (0.80), SAD (0.77), GAD (0.84), OCD (0.72), MD (0.76), and total score (0.92).

The *Specific Phobia subscale of the Spence Children's Anxiety Scale; SCAS* (Spence, 1997) consists of five items with four Likert alternatives (0 = *never*, 3 = *always*). We used the Spanish version, which had an average internal consistency reliability of 0.64 (Orgiles et al., 2016). The McDonald's ω for our sample was 0.62.

The *Children's Revised Impact of Event Scale (CRIES*; Yule, 1997) is a screening scale of PTSD for children over 8 years old. It consists of eight items rated on four-point Likert scale (0–3) and provides two subscales, four items assessing trauma-related intrusion and four avoidance. In this sample, the McDonald's ω were as follows: Intrusion (0.88), Avoidance (0.84), and total score (0.91).

#### Subjective Well-Being

The *Mental Health Inventory* (MHI; Veit and Ware, 1983) is a 38-item measure of psychological distress and well-being, developed for use in general populations, and responded from 0 (never) to 3 (always). Its factor structure for adults is psychological distress (anxiety, depression, and loss of behavioral emotional control) and psychological well-being, with general positive affect and emotional ties as subscales. Our own preliminary data from a study in progress indicate that its factor structure consists of two factors: distress (23 items) and well-being (15 items). The internal consistency (McDonald's ω) for both subscales in this sample was 0.93 and 87, respectively.

The Revised Mental Health Inventory-5 (*MHI-5;* Rivera-Riquelme et al., 2019) is a useful instrument to assess mental health bidimensionally, as well as to detect anxiety and depression symptoms in children and adolescents. The original MHI-5 is a brief, valid, and reliable international instrument for assessing mental health in adults (Berwick et al., 1991) as well as in children and adolescents (Marques et al., 2011). The revision of original MHI-5 consisted of the adaptation of the response format to four choices (0 = *never*, 1 = *sometimes*, 2 = *often*, and 3 = *always*). Higher scores indicate better mental health. The Revised MHI-5 has shown good psychometric properties similar to previous studies in different cultures and populations (Rivera-Riquelme et al., 2019). In this sample, we reported McDonald's ω of 0.73.

#### Suicidal-Related Behaviors

We used items 21 and 28 from the MHI to assess suicide-related behaviors: “During the past month, how often have you felt that others would be better off if you were dead?” and “During the past month, did you think about taking your own life?” These questions are answered in a Likert-type response scale from 0 (never) to 3 (always).

### Procedure

The procedure we followed for validation of the DetectaWeb-Distress Scale was divided into four phases: (1) development of a web-based application for administration; (2) development of the instrument, according to steps for test development by (Muñiz and Fonseca-Pedrero, 2019); (3) application of the new questionnaire to a community sample; and (4) data analysis. The description of the complete procedure of the development of the instrument as well as of the DetectaWeb Project can be found elsewhere (Garcia-Olcina et al., 2014; Piqueras, Garcia-Olcina et al., 2017). DetectaWeb Project is a web-based early detection program of mental health rated on a continuum, which assesses psychological distress (DetectaWeb-Distress Scale) as well as psychological well-being (DetectaWeb-Well-being Scale) in children and adolescents. This web-based assessment protocol from the Bidimensional Mental Health Model (BMHM) has also been employed in two previous studies (Piqueras et al., 2019; Rivera-Riquelme et al., 2019).

### Ethical Considerations

This study obtained the approval of the Ethical Committee for research projects (Órgano Evaluador de Proyectos, OEP) from the Vice-Rectory for Research and Technological Development of the Miguel Hernandez University (reference numbers DPS-JPR-001-10 and DPS.JPR.02.14). Children over 12 years old and their parents were requested to provide informed consent.

### Statistical Analysis

All analyses were conducted with SPSS 24, EQS 6.3, and FACTOR 10.4. First, we examined item distribution and frequencies of the items. Previously, the analysis of outliers was carried out by graphically representing the results (box diagrams). Although outliers were detected, it was decided not to remove them from the sample for reasons of ecological validity. Concerning missing values, we did not have any of them, as it was mandatory to answer all the questions in order to finish the online survey.

Next, we tested a model (Model B) with nine correlated factors, a model found in the exploratory factor analysis (EFA) of the preliminary data with an adolescent sample (Garcia-Olcina et al., 2014). Then, other alternative models were examined: (i) Model A: all 30 items grouped into one general factor; (ii) Model B: nine correlated factors grouped into depression (MD and DD) and S, GAD, SoPh, SAD, SP, Pd/Ag, PTSD, and OCD; (iii) Model C: 10 correlated factors with three items per scale; (iv) Model D: 10 factors (Model C) grouped under one second-order factor that corresponds to the total scale; (v) Model E: a DSM5-based model with10 first-order factors grouped into 5 correlated second-order factors [depression (DD and D), S, anxiety (SAD, SoPh, SP, Pd/Ag, and GAD), OCD, and PTSD]; (vi) Model F: a DSM5-based model with 10 first-order factors (Model E) grouped into 5 second-order factors plus a general third-order factor (total scale).

We used correlation matrices and the Robust maximum likelihood (ML) method in all cases (EQS 6.3). We calculated the following indices as goodness-of-fit measures: Satorra-Bentler chi-square; S-B χ^2^/degrees of freedom (χ^2^/*df*) ratio (Chau, 1997); Root Mean Square Error of Approximation (RMSEA) (Schumacker and Lomax, 2004); Standardized Root Mean Square Residual (SRMR), Comparative Fit Index (CFI), Goodness of Fit Index (GFI), Adjusted Goodness of Fit Index (AGFI) (Bentler, 1990), and Akaike Information Criterion (AIC) (Jöreskog and Sörbom, 1993).

Later, we tested whether the DetectaWeb-Distress Scale exhibits metric invariance. Progressive evaluation of Factor Invariance (FI) was conducted through the Mean and Covariances Structures (MACS) method, as recommended by Byrne et al. (2009). The reported fit indices were RMSEA and CFI, which are the main indicators to evaluate FI. According to Cheung and Rensvold (2002), invariance between samples is admissible when the difference between the CFIs (Increment CFI) is ≤0.01 with respect to the previous model. The CFI is complemented by the AIC, which is interpreted as absence of FI when there is a considerable increase in this index. Mardia (1974) test was employed to assess multivariate normality of data, in which values lower than 5.00 are indicative of normality. The estimation method used was Robust ML.

When there is strong measurement invariance, the comparison of factor means across groups is permissible (Dimitrov, 2012). Consequently, we calculated age and gender differences. We also estimated Cohen (1988) *d* index (standardized mean difference), which allows evaluating the effect size (ES) of the obtained differences. McDonald (1999) ω was used to estimate the internal consistency of the DetectaWeb-Distress total scale and subscales; it is a better estimator of reliability than Cronbach's α (Dunn et al., 2014).

Finally, convergent-discriminant validity was evaluated by calculating the correlation coefficients between the scores on the DetectaWeb-Distress Scale and different well-established measures. Cohen's criteria were used to estimate the ES of the correlations (Cohen, 1988; Lipsey and Wilson, 2001).

## Results

### Item Analysis and Reliability

The frequencies of item responses indicated that all response options had been chosen. Items 7, 8, and 9 (S) obtained less frequent responses of “often” and “always.”

The mean item response ranged from 0.07 (Item 9) to 2.11 (Item 22), and standard deviations ranged from 0.34 (Item 9) to 1.08 (Item 11). The mean response of the items was 0.71 (SD = 0.37), which is noticeably lower than the average theoretical point of the scale, 1.5. With respect to the values of the correlations, the item total of corresponding subscale corrected index did not find any value <0.30 (${\text{r}}_{\text{it}}^{\text{c}}$) (Nunnally, 1994) (see Table 2).

**Table 2:** Means (M), standard deviation (SD), corrected item total of corresponding subscale correlation (${\text{r}}_{\text{it}}^{\text{c}}$), Cronbach α if item eliminated (α-i), and reliability (McDonald's ω) of the Detectaweb-Distress Scale.

**Items**	**M**	**SD**	**${\text{r}}_{\text{it}}^{\text{c}}$**	**α-i**	**McDonald's ω**	**Standardized factor loadings**
**MD**						
1. Depressed or very sad	0.66	0.69	0.51	0.72	0.75	0.67
2. Less interested in doing activities	0.88	0.84	0.38	0.72		0.48
3. Think one's is not worth anything	0.39	0.72	0.45	0.72		0.68
**DD**						
4. Feel more days sad/down than good	0.66	0.72	0.44	0.72	0.67	0.66
5. Feel like doing nothing	0.75	0.73	0.33	0.72		0.51
6. Find it harder than usual to have fun	0.53	0.81	0.33	0.72		0.49
**S**						
7. Thoughts of taking your own life	0.17	0.48	0.77	0.73	0.94	0.90
8. Thoughts of ways to take your life	0.17	0.48	0.72	0.73		0.80
9. Attempts to take your life	0.07	0.34	0.61	0.73		0.65
**SAD**						
10. Afraid to be away from parents	0.66	0.85	0.45	0.72	0.71	0.64
11. Worried about something bad will happen to parents	2.04	1.08	0.31	0.72		0.45
12. Afraid to stay home alone	0.49	0.78	0.38	0.72		0.62
**SoPh**						
13. Fear of negative evaluation	1.05	0.97	0.54	0.72	0.79	0.66
14. Fear of people can laugh at you	0.80	0.86	0.61	0.72		0.77
15. Feel worried about feeling embarrassing situations	0.95	0.94	0.54	0.72		0.66
**SP**						
16. Animal phobias	0.74	0.99	0.36	0.72	0.65	0.54
17. Blood/injury/injections phobias	0.68	0.97	0.40	0.72		0.57
18. Situational phobias	0.44	0.73	0.34	0.72		0.52
**Pd/Ag**						
19. Get suddenly frightened without apparent reason	0.58	0.70	0.52	0.72	0.78	0.69
20. Worried about feeling suddenly terrified	0.50	0.72	0.56	0.72		0.72
21. Fear of places where feeling sudden fear without possibility of escaping / being helped	0.51	0.75	0.39	0.72		0.54
**GAD**						
22. Worry a lot about things like school, your friends, etc.	2.11	0.95	0.42	0.72	0.67	0.51
23. Worry about some things more than other peers	1.23	0.96	0.43	0.72		0.57
24. Worry about future	1.53	1.06	0.38	0.72		0.63
**OCD**						
25. Thoughts or images which seem absurd or meaningless, but that frighten or bother you	0.70	0.76	0.32	0.72	0.62	0.56
26. Repeating thoughts about getting contaminated	0.38	0.66	0.33	0.72		0.48
27. Need for repeating some actions over and over again, even if it seems absurd	0.54	0.83	0.31	0.72		0.47
**PTSD**						
28. Experience a stressful or traumatic event	0.38	0.69	0.47	0.72	0.75	0.59
29. Experience, witness or have to deal with a stressful/traumatic event	0.33	0.62	0.50	0.73		0.59
30. After experiencing or witnessing such a stressful/traumatic event, feeling symptoms such as unwanted thoughts, nightmares, etc.	0.42	0.73	0.48	0.72		0.72
Overall Distress	21.30	11.05	1.00	0.87	0.91	

*MD, major depression; DD, dysthymic disorder; S, suicidality (suicidal ideation, plans, and attempts); SAD, separation anxiety disorder; SoPh, social phobia; SP, specific phobia; Pd/Ag, panic disorder/agoraphobia; GAD, generalized anxiety disorder; OCD, obsessive–compulsive disorder; PTSD, post-traumatic stress disorder (PTSD); Overall Distress, total score indicating global distress or emotional symptomatology; Standardized factor loadings, these values show the degree of relationship (factor loadings) for each item on its corresponding factor resulting from the confirmatory factor analysis for Model C (10 correlated factors)*.

### Confirmatory Factor Analysis (CFA)

As can be seen in Table 3, goodness-of-fit indices indicated that the best models were Models B and C, with CFI, GFI, and AGFI equal to or >0.90, and RMSEA < 0.05. Models A and D did not receive empirical support: in Model A, RMSEA was >0.60, and in both cases, CFI was <0.85. The other models showed adequate fit indices, even the DSM5-based models (E and F), due to the fact that the goodness-of-fit indices indicated that these models fit the data acceptably. However, Model C had the best fit indices as well as corresponding the best with the theoretical model we considered (10 different but correlated emotional disorders).

**Table 3:** Goodness-of-fit indices of confirmatory factor analysis.

Models^*^	χ^2^^***^	**df**	**χ^2/^df**	**RMSEA [90% CI]**	**CFI**	**SRMR**	**AIC**	**GFI**	**AGFI**
Model A	3436.48	405	8.48	0.07 [0.06,0.07]	0.57	0.08	2626.48	0.76	0.73
Model B	954.50	369	2.58	0.03 [0.03,0.03]	0.92	0.04	216.59	0.94	0.93
Model C^**^	**929.16**	**360**	**2.58**	**0.03 [0.03,0.03]**	**0.92**	**0.04**	**209.16**	**0.95**	**0.93**
Model D	1449.15	394	3.67	0.04 [0.04,0.04]	0.85	0.06	661.15	0.91	0.89
Model E	1279.56	389	3.29	0.04 [0.04,0.04]	0.87	0.05	501.56	0.93	0.91
Model F	1095.23	390	2.81	0.03 [0.03,0.04]	0.90	0.05	315.23	0.93	0.92

* *Model A, single factor; Model B: nine correlated factors; Model C, 10 correlated factors; Model D, Model C plus one second-order factor; Model E, DSM5-based model with 10 first-order plus 5 second-order correlated factors; Model F, Model E plus a third-order factor*.

** *The model with the best fit shown in bold*.

*** *Satorra–Bentler Chi Square*.

Table 2 (last column) shows the degree of relationship (standardized weights) for each item on its corresponding factor. All item weights are above or near 0.60 (0.45–0.90).

### Factor Invariance

First, the FI of Model C was tested across age (see Table 4). Mardia's test indicated non-normal data. Configural, weak, and strong invariance models were tested using Robust ML. The configural invariance model fit adequately with Robust ML due to the fact that the CFI was 0.91, which is larger than 0.90 (Schermelleh-Engel et al., 2003). The weak invariance model also showed an adequate fit, because the change in the CFI was not >0.01, and the AIC increased slightly. Next, the strong invariance model was also confirmed, because the change in the CFI was <0.01, and the AIC increased only slightly. Finally, the strict FI model did not fit adequately, because the reduction of the CFI was higher than 0.01, and the AIC increased considerably.

**Table 4:** Fit indices of invariance models across age and gender.

**Invariance model**	χ^2^^*^	**df**	**χ^2/^df**	**RMSEA [90% CI]**	**CFI**	**AIC**
**Invariance across age**						
Configural Invariance	1327.57	720	1.84	0.034 [0.031,0.036]	0.914	−112.42
Weak Invariance	1366.03	740	1.84	0.034 [0.031,0.036]	0.911	−113.96
Strong Invariance	1707.07	760	2.24	0.035 [0.032,0.038]	0.910	187.07
Strict Invariance	1905.13	780	2.44	0.039 [0.036,0.042]	0.884	345.13
**Invariance across gender**						
Configural Invariance	1286.93	720	1.78	0.032 [0.030,0.035]	0.916	−153.06
Weak Invariance	1304.00	740	1.76	0.032 [0.029,0.035]	0.916	−175.99
Strong Invariance	1463.98	760	1.92	0.033 [0.030,0.035]	0.917	−56.01
Strict Invariance	1600.09	780	2.05	0.035 [0.033,0.038]	0.901	40.10

*χ^2^, Satorra–Bentler's Chi Square; df, degree of freedom; RMSEA [90% CI], root mean square error of approximation with 90% confidence interval*.

Second, we tested the FI across gender (see Table 4). The configural invariance model fit adequately due to a CFI value of 0.916, which indicates adequate fit (Schermelleh-Engel et al., 2003). The weak invariance model showed the same CFI, and the AIC did not increase considerably. The analysis of strong invariance indicated adequate fit because the CFI did not change, but the AIC increased slightly. Lastly, the strict FI model was tested and showed that the CFI decreased around 0.01, and the AIC increased.

### Gender and Age Differences on the DetectaWeb-Distress Scale

Table 5 shows the means, standard deviations, and scale scores based on gender and age. Overall, girls scored higher than boys, but the differences were significant only for MD, SAD, SP, Pd/Ag, GAD, and OCD. Regarding age, children scored higher than adolescents on SAD, SP, Pd/Ag, and OCD, whereas adolescents displayed higher scores on MD, DD, S, and GAD. The ESs were small for all comparisons with the exception of SP, which reached a medium magnitude, with higher scores for females (*d* = 0.51).

**Table 5:** Means and standard deviations of DetectaWeb-Distress Scale scores based on gender and age.

**Gender**	**Females (*****n*** **= 745)**		**Males (*****n*** **= 754)**		***p***	***d***
	**M**	**SD**	**M**	**SD**		
MD	2.08	1.71	1.77	1.70	^***^	0.18
DD	1.99	1.68	1.85	1.63	-	0.08
S	0.41	1.11	0.40	1.15	-	0.01
SAD	3.65	2.06	2.73	1.83	^***^	0.47
SoPh	3.03	2.23	2.54	2.17	^***^	0.22
SP	2.35	2.07	1.37	1.74	^***^	0.51
Pd/Ag	1.85	1.78	1.31	1.55	^***^	0.32
GAD	5.23	2.15	4.48	2.26	^***^	0.34
OCD	1.71	1.64	1.52	1.57	^*^	0.12
PTSD	1.11	1.60	1.15	1.59		−0.02
Total	23.46	10.92	19.18	10.77	^***^	0.39
**Age**	**Children 8–12 years (*****n*** **= 689)**		**Adolescents 13–18 years (*****n*** **= 810)**		***p***	***d***
	**M**	**SD**	**M**	**SD**		
MD	1.58	1.58	2.22	1.76	^***^	−0.38
DD	1.69	1.66	2.12	1.63	^***^	−0.26
S	0.31	1.12	0.49	1.13	^**^	−0.16
SAD	3.59	2.16	2.83	1.78	^***^	0.38
SoPh	2.88	2.32	2.71	2.11	-	0.08
SP	2.04	2.06	1.69	1.89	^***^	0.18
Pd/Ag	1.80	1.80	1.39	1.56	^***^	0.24
GAD	4.57	2.41	5.10	2.04	^***^	−0.24
OCD	1.84	1.73	1.43	1.48	^***^	0.25
PTSD	1.21	1.69	1.06	1.51	-	0.09
Total	21.56	11.99	21.09	10.19	-	0.04

*MD, major depression; DD, dysthymic disorder; S, suicidality (suicidal ideation, plans, and attempts); SAD, separation anxiety disorder; SoPh, social phobia; SP, specific phobia; Pd/Ag, panic disorder/agoraphobia; GAD, generalized anxiety disorder; OCD, obsessive–compulsive disorder; PTSD, post-traumatic stress disorder (PTSD); Overall Distress, total score indicating global distress or emotional symptomatology*.

* p < 0.05;

** p < 0.01;

*** *p < 0.001*.

### Estimations of Reliability

The reliability estimations were calculated with McDonald's ω. The reliability for the overall distress score was 0.91. The remaining values were between 0.65 and 0.94 (see Table 2). Moreover, as the DSM5-based factorial model showed a good fit, the calculation and use of total scores for depressive symptoms (MD + DD) and anxiety symptoms (sum of SAD, GAD, SoPh, Pd/Ag, and SP) was justified, which resulted in an internal consistency of 0.82 and 87, for depressive and anxiety symptoms, respectively.

### Convergent-Discriminant Validity

We analyzed the bivariate correlations (Pearson's coefficients) between subscale scores of the DetectaWeb-Distress Scale and other patient-reported outcome measure for internalizing problems (Table 6). Significant and positive relationships between all scores were found (*p* < 0.01). First, correlations between the DetectaWeb-Distress subscale scores ranged from 0.43 (S) to 0.71 (SoPh), with a general trend to find a lower relationship of S with the other subscales (*r* = 0.06–0.43) than for other associations (0.17–0.59). Second, concerning the relationship between the RCADS-30 and the DetectaWeb-Distress Scale scores, correlation coefficients ranged between 0.35 and 0.82. Third, the relationships between the score on DetectaWeb-Distress SP subscale and the equivalent SCAS and SP subscale were positive, and the PTSD subscale of the DetectaWeb-Distress Scale and the CRIES scores were also positively correlated. Finally, the score on S of the DetectaWeb-Distress Scale positively correlated with items related to S and the Distress score of the MHI, as well as negatively with well-being of the MHI and the MHI-5 (see Table 6).

**Table 6:** Convergent-discriminant validity of the DetectaWeb-Distress Scale.

**PROM**		**DetectaWeb-Distress Scale**										
		**MD**	**DD**	**S**	**SAD**	**SoPh**	**SP**	**Pd/Ag**	**GAD**	**OCD**	**PTSD**	**OD**
DetectaWeb-Distress Scale	D	0.58^**^										
	S	0.43^**^	0.31^**^									
	SA	0.18^**^	0.17^**^	0.06^*^								
	SoPh	0.41^**^	0.35^**^	0.22^**^	0.38^**^							
	SP	0.24^**^	0.25^**^	0.12^**^	0.36^**^	0.35^**^						
	Pd/A	0.34^**^	0.31^**^	0.22^**^	0.43^**^	0.43^**^	0.43^**^					
	GA	0.29^**^	0.25^**^	0.12^**^	0.39^**^	0.40^**^	0.25^**^	0.32^**^				
	OC	0.33^**^	0.28^**^	0.23^**^	0.34^**^	0.35^**^	0.33^**^	0.46^**^	0.33^**^			
	PTS	0.30^**^	0.28^**^	0.29^**^	0.22^**^	0.25^**^	0.22^**^	0.34^**^	0.20^**^	0.41^**^		
	Total	0.65^**^	0.60^**^	0.43^**^	0.61^**^	0.71^**^	0.60^**^	0.70^**^	0.62^**^	0.65^**^	0.55^**^	
RCADS-30	MDR	0.67^**^	0.64^**^	0.34^**^	0.23^**^	0.44^**^	0.30^**^	0.38^**^	0.33^**^	0.42^**^	0.34^**^	0.66^**^
	PR	0.44^**^	0.39^**^	0.37^**^	0.33^**^	0.34^**^	0.31^**^	0.53^**^	0.25^**^	0.47^**^	0.45^**^	0.62^**^
	SoPhR	0.40^**^	0.37^**^	0.16^**^	0.37^**^	0.67^**^	0.35^**^	0.40^**^	0.41^**^	0.41^**^	0.28^**^	0.65^**^
	SADR	0.17^**^	0.17^**^	0.13^**^	0.65^**^	0.34^**^	0.40^**^	0.45^**^	0.23^**^	0.38^**^	0.32^**^	0.54^**^
	GADR	0.22^**^	0.18^**^	0.06^*^	0.54^**^	0.40^**^	0.32^**^	0.39^**^	0.60^**^	0.42^**^	0.22^**^	0.58^**^
	OCDR	0.33^**^	0.31^**^	0.24^**^	0.37^**^	0.39^**^	0.31^**^	0.45^**^	0.36^**^	0.62^**^	0.40^**^	0.62^**^
	TotalR	0.48^**^	0.44^**^	0.27^**^	0.57^**^	0.59^**^	0.45^**^	0.58^**^	0.52^**^	0.60^**^	0.43^**^	0.82^**^
SCAS	SPS	0.23^**^	0.22^**^	0.11^**^	0.35^**^	0.32^**^	0.63^**^	0.37^**^	0.23^**^	0.30^**^	0.20^**^	0.49^**^
CRIES	Intrusion	0.36^**^	0.30^**^	0.22^**^	0.21^**^	0.27^**^	0.19^**^	0.28^**^	0.27^**^	0.33^**^	0.37^**^	0.45^**^
	Avoidance	0.26^**^	0.22^**^	0.15^**^	0.24^**^	0.26^**^	0.18^**^	0.27^**^	0.27^**^	0.27^**^	0.30^**^	0.40^**^
	Total	0.33^**^	0.28^**^	0.20^**^	0.24^**^	0.28^**^	0.20^**^	0.29^**^	0.29^**^	0.32^**^	0.35^**^	0.45^**^
MHI suicide	Distress	0.72^**^	0.61^**^	0.45^**^	0.21^**^	0.44^**^	0.26^**^	0.43^**^	0.34^**^	0.42^**^	0.36^**^	0.71^**^
	Item 21	0.49^**^	0.41^**^	0.53^**^	0.08	0.32^**^	0.12^**^	0.23^**^	0.13^**^	0.22^**^	0.30^**^	0.45^**^
	Item 28	0.32^**^	0.27^**^	0.60^**^	0.06	0.18^**^	0.07	0.08	−0.024	0.14^**^	0.29^**^	0.30^**^
	Well-being	−0.54^**^	−0.49^**^	−0.31^**^	−0.06	−0.31^**^	−0.16^**^	−0.18^**^	−0.11^**^	−0.22^**^	−0.19^**^	−0.44^**^
MHI-5	Well-being	−0.57^**^	−0.50^**^	−0.32^**^	−0.12^**^	−0.32^**^	−0.20^**^	−0.30^**^	−0.22^**^	−0.27^**^	−0.26^**^	−0.49^**^

PROM, Patient-Reported Outcome Measures; DetectaWeb, Distress Scale; MD, major depression; DD, dysthymic disorder; S, suicidality (suicidal ideation, plans, and attempts); SAD, separation anxiety disorder; SoPh, social phobia; SP, specific phobia; Pd/Ag, panic disorder/agoraphobia; GAD, generalized anxiety disorder; OCD, obsessive–compulsive disorder; PTSD, post-traumatic stress disorder (PTSD); Total, Overall Distress, total score indicating global distress or emotional symptomatology; RCADS-30, Revised Childhood Anxiety and Depression Scale-30 items; MDR, major depression; DDR, dysthymic disorder; PRR, Panic disorder; SoPhR, social phobia; SADR, separation anxiety disorder; GADR, generalized anxiety disorder; OCDR, obsessive–compulsive disorder; TotalR, Overall Distress, total score indicating global distress or emotional symptomatology; SCAS, Children's Spence Anxiety Scale; SPS, Specific Phobia; CRIES, Children's Revised Impact of Event Scale; MHI, Mental Health Inventory; Item 21, During the past month, how often have you felt that others would be better off if you were dead?; Item 22, During the past month, did you think about taking your own life?; MHI-5, Mental Health Inventory-5 items;

* p < 0.05;

** *p < 0.01*.

## Discussion

The aim of this study was to examine the the reliability and different sources of validity evidence of the DetectaWeb-Distress Scale in children and adolescents.

First, an item analysis was performed, which showed that the mean scores of the items were adequate, as they were close to the midpoint of the scale. In addition, all response options for the items were chosen, with a limited range of responses for those items that corresponded to the suicidality factor. Concerning item total of corresponding subscale correlations, we did not find any value lower than 0.30.

The CFA tested the nine-factor model reported in previous studies (Garcia-Olcina et al., 2014; Piqueras et al., 2020), as well as other theory-based models. However, the best fit to data was for the 10 correlated-factor model, which included MD, DD, S, SAD, SoPh, SP, Pd/Ag, GAD, OCD, and PTSD. The explanation for this finding is that, in this study, the factor of depressive disorders was divided into two related disorders: MD and DD. Our model is equivalent to other measures reporting multidimensional models where each factor corresponds to the dimension that it aims to measure (e.g., RCADS or SCAS). However, our instrument includes more emotional disorder symptoms with a lower number of items, showing equivalent psychometric properties to previous measures. Anyway, a more detailed discussion of the model or models that appear to underlie the instrument in comparison to other possible models that have not been tested in the present study deserves some mention.

So, on the one hand, it seems that our results also support two DSM5-based models tested in the study (Models D and E) and, therefore, they seem to support the DSM-5 proposal for the existence of a suicidal behavior disorder, since suicidal symptoms form a distinct factor from other emotional symptoms, although a related factor. However, it would be possible that a model that included depressive and suicidal symptoms within the same factor would also fit the data well. This model has not been tested in the present study and should await further research. In this sense, it must be remembered that the proposal of a suicidal behavior disorder is a controversial issue, and in fact, in the DSM-5, it was included in the chapter dedicated to conditions for further study.

On the other hand, from a theoretical point of view, the psychological theoretical model that could framework our finding could be the accumulative tradition of alternative proposal focused on solving the shortcomings of traditional taxonomies in the form of a quantitative nosology, an evidence-based organization of psychopathology (e.g., Krueger, 1999; Achenbach and Rescorla, 2001, 2007; Kessler et al., 2002; Watson, 2005; Clark and Watson, 2006; Kotov et al., 2017). These quantitative nosologies, rather than being constructed from the top down, have emerged from the independent work of multiple research groups trying to understand the natural organization of psychopathology (Kotov et al., 2017). According to this HiTOP model, the dimensions or spectra that make it possible to obtain the super spectra or high-order dimensions are six (spectra). They are established to categorize the different subfactors of symptoms. The so-called internalizing spectrum includes sexual problems, eating pathology, fear, distress, and mania (subfactors). Fear subfactor includes SoPh, Pd/Ag, SP, SAD, and OCD, while Distress subfactor includes MD, DD, GAD, PTSD, and borderline personality disorder. All these syndromes or disorders emerge from symptom components and maladaptive traits (Components) and symptoms (Signs and Symptoms) (see Figure 2. Spectra of the Hierarchical Taxonomy of Psychopathology in Kotov et al., 2017).

Concerning the factorial invariance analyses with the MACS method, in general, our results showed factor structure equivalence across age and gender. Consequently, it allows us not only to compare mean scores of items and factors but also to conclude that the factor structure is equivalent in both groups (Dimitrov, 2010). In summary, strong measurement invariance was found for both gender and age variables, which indicates equal factor loadings and equal indicator intercepts (i.e., indicator means) across groups. This finding implies that, when strong measurement invariance is shown, the comparison of factor means across groups is permissible (Dimitrov, 2012).

Regarding gender and age differences, we found that girls scored higher than boys on most of the subscales, which is consistent with previous studies (Garcia-Olcina et al., 2014; Lewis et al., 2019). In terms of age, children had higher scores for SAD, SP, Pan/Ag, and OCD symptoms, whereas adolescents scored higher on MD, DD, S, and GAD. These findings are consistent with previous studies that point out that age is a conditioning factor of the different anxiety and depression symptoms (Canals et al., 2019). These results are also compatible with the HiTOP Model, which would suggest that the Fear subfactor (SoPh, Aga, SP, SAD, PD, and OCD) would have an earlier onset, while the Distress subfactor (MD, DD, GAD, and PTSD) would be more prevalent among adolescents (Kotov et al., 2017).

Internal consistency reliability for the Overall Distress Scale (0.91) was higher than the 0.70 recommended by Nunnally (1994). This reliability value is equivalent to those reported in previous studies using web-based measures of anxiety and depression with internal consistencies between 0.88 and 0.95 (van Ballegooijen et al., 2016), as well as the reliability reported for the RCADS and SCAS (mean α values of 0.93 and 0.92, respectively). The DetectaWeb-Distress subscales obtained values between 0.62 and 0.94. These values are equivalent to those reported for measures of anxiety and depression such as the RCADS, ranging from 0.74 to 0.85 (Piqueras et al., 2017b), and the SCAS, ranging from 0.64 to 0.80 (Orgiles et al., 2016).

As for evidence of validity, our results showed good convergent-discriminant validity, with significant correlations between the DetectaWeb-Distress Scale and other measures of related constructs. The correlation with the RCADS was *r* = 0.82, which is consistent with other results reported with measures of anxiety and depression symptomatology in youth, such as the Epidemiological Studies Depression Scale (CESD) and the Major Depression Inventory (MDI) with correlations of 0.88, or the Depression and Anxiety subscales of the Depression, Anxiety, and Stress Scale (DASS), with a correlation of 0.83 (Cuijpers et al., 2008; Zlomke, 2009). Additionally, correlations between the DetectaWeb-Distress Scale subscales and the RCADS subscales were significant, showing ESs between moderate and large (*r* = 0.35–0.67). These findings are consistent with those reported in previous studies. For example, Zlomke (2009) found moderate correlations between the DASS scales and the Penn State Worry Questionnaire (PSWQ), ranging from 0.28 to 0.49.

The DetectaWeb-Distress Scale subscales that had no analog dimensions in the RCADS also correlated strongly with other specific measures that we included. SP showed a high correlation with the homolog subscale of the SCAS (*r* = 0.63). This finding was similar to that of Orgiles et al. (2012), who obtained a moderate but significant correlation between the SCAS subscale for Physical Injury Fears and the STAI-C. Our subscale of PTSD symptoms showed a significant moderate correlation with the CRIES total score (*r* = 0.35), which coincides with the study by Zlomke (2009) in which there was a correlation of 0.49 between the Stress subscale of the DASS and the PSWQ. We also found a high correlation between the S subscale and the MHI items for suicide.

In summary, our first hypothesis concerning the correlated 10-factor solution was supported. The second hypothesis was confirmed: factorial invariance across gender and age was revealed. The third hypothesis was also confirmed, as we obtained age and gender differences in the symptom subscales, as expected. With regard to our fourth hypothesis, we obtained good internal consistency reliability, which is equivalent to the results of other web-based questionnaires (van Ballegooijen et al., 2016). Finally, the fifth hypothesis was confirmed due to the fact that correlations with equivalent measures that assess the same or related constructs were significant and positive.

Some methodological limitations should be noted. First, this scale seems to have reliable and valid indicators for emotional symptomatology in youth, but it is not up to date with the current 5th edition of the DSM (DSM-5, American Psychological A.ssociation, 2013). DSM-5 has made two main changes with regard to the anxiety disorders section: (1) selective mutism is now included as an anxiety disorder, and (2) OCD and PTSD have been removed from the section as they are no longer considered as pure anxiety syndromes. However, the DetectaWeb-Distress Scale would allow the calculation of different scores compatible with the current DSM-5: a general indicator of depressive disorder symptoms and specific symptoms of unipolar mood disorders (MD and DD), a total index of anxiety disorder symptoms plus each specific symptomatology (SAD, GAD, SoPh, Pd/Ag, SP), as well as OCD, PTSD, and S indices, which can be considered anxiety and depression-related disorders following DSM-5 rationale. Second, this study should be extended to clinical samples in order to provide clinical validity and to allow clearly differentiating between healthy and anxious/depressive children and adolescents, although a recent study addressed this issue (Piqueras et al., 2020). Third, according to the conceptualization of mental health as a continuum of psychological distress and well-being, future studies should provide data concerning both poles of the mental health continuum. Fourth, the DetectaWeb-Distress scores should be compared with clinical diagnostic interviews in order to examine diagnostic validity, also tested in Piqueras et al. (2020). Anyway, it should be mentioned that this measure may be useful to screen and detect, but it is not a tool to diagnose emotional disorders. Fifth, cross-cultural studies are needed to determine the psychometric properties of the scale across languages and cultures. It is expected that these findings may be generalized to non-Spanish-speaking children and adolescents (i.e., data should be replicated with other Latino groups from Latin America or USA, as well as with English-speaking participants). Finally, the present study has got some other constraints typically found with the use of self-report measures, such as the convenience of using other methods to generalize our findings (e.g., multiple informants) and the absence of social desirability scales or of infrequency scales to detect random responses, among other bias. Despite these limitations, we note that this measure has several strengths, such as its brevity and being one of the first measures developed specifically for online use.

## Conclusions

In summary, support was found for the DetectaWeb-Distress Scale as a valid and useful web-based instrument for the early screening and identification of anxiety and depression and some of the more common related emotional symptoms (depression, OCD, or PTSD), as well as for suicidality, in children and adolescents. It is the first one specifically developed for use through the Internet. Furthermore, the scale has potential as a useful instrument in the implementation of preventive interventions for anxiety–depression and related symptoms, as well as for the promotion of well-being and mental health.

## Data Availability Statement

The raw data supporting the conclusions of this article will be made available by the authors, without undue reservation.

## Ethics Statement

The studies involving human participants were reviewed and approved by Ethical Committee for research projects (Órgano Evaluador de Proyectos, OEP) from the Vice-Rectory for Research and Technological Development of the Miguel Hernandez University (reference numbers DPS-JPR-001-10 and DPS.JPR.02.14). Written informed consent to participate in this study was provided by the participants' legal guardian/next of kin.

## Author Contributions

JP conceived the study, participated in the data collection, led the preparations, analyzed the data, and wrote the first draft of the manuscript. MG-O, MR-R, and AM-G participated in the recruitment of the sample and data collection. PC participated in the study conception and in the data analysis. All authors contributed to interpreting the data, helped to draft and revise the manuscript, and read and approved the final manuscript.

## Conflict of Interest

The authors declare that the research was conducted in the absence of any commercial or financial relationships that could be construed as a potential conflict of interest.
